# Determining the optimal level of buffel grass hay as a source of physically effective fiber in a diet with high proportions of forage cactus for sheep finished in the feedlot

**DOI:** 10.1007/s11250-025-04834-5

**Published:** 2026-04-11

**Authors:** Diego Francisco Oliveira Coelho, Juliana Silva de Oliveira, João Paulo de Farias Ramos, Danillo Marte Pereira, Alberto Jefferson da Silva Macêdo, Celso José Bruno de Oliveira, Mateus Lacerda Pereira Lemos, Nelquides Braz Viana, Paloma Gabriela Batista Gomes, Guilherme Medeiros Leite, Edson Mauro Santos

**Affiliations:** 1 Department of Animal Science, Federal University of Paraíba, Areia, Paraíba, 58.397-000 Brazil; 2 Department of Animal Science, Federal University of Maranhão, Chapadinha, Maranhão, 65.500-000 Brazil

**Keywords:** Buffel grass hay, Consumption, Digestibility, Economic indicators, Sheep, Weight gain

## Abstract

The objective was to determine the optimum level of buffel grass hay (*Cenchrus ciliaris* L.) as a source of physically effective fiber in diets with high proportions of forage cactus on the bioeconomic performance of sheep finished in a dryland feedlot. A completely randomized experimental design was employed, consisting of four treatments and ten replications, with treatments defined by the levels of buffel grass hay (45, 30, 15, and 7.5% of buffel grass hay in the diet’s dry matter). A quadratic effect was noted for intakes of organic matter, dry matter, crude protein, ether extract, neutral detergent fiber, non-fibrous carbohydrates, total carbohydrates, total digestible nutrients, and metabolizable energy, with maximum intakes estimated at 35, 42, 23, 20, 25, 10, 25, and 24% of buffel grass hay, respectively. A linear effect was observed for all apparent digestibility variables. A quadratic effect was identified for final weight, total gain, and average daily gain, with maximum gains estimated by the regression model at levels of 27, 27, and 28% buffel grass hay, respectively. Based on productive performance and net income achieved in feedlot, the inclusion of 30% buffel grass hay in diets with high proportions of forage cactus is recommended for sheep in the feedlot.

## Introduction

Due to the soil and climate conditions in drylands, meat or milk production systems are characterized by low production rates, making them vulnerable to variations in forage availability, both in quantity and quality. Currently, the viability of these production systems is associated with the ability to grow forage cactus.

Forage cactus stands out for its high biomass production potential, with productivity variations of 10 and 40 t ha^-^¹ of dry matter (DM) under dryland conditions (Pastorelli et al. 2022). In addition, its high moisture content, which can reach 900 g kg (Pereira et al. 2021), contributes to livestock desiccation during the dry period of the year, making it a strategic resource in drylands.

From a nutritional point of view, forage cactus is a source of non-fibrous carbohydrates (NFC) (± 600 g kg DM), making it an important energy component in ruminant feed. However, it has low concentrations of neutral detergent insoluble fiber (NDF) (± 250 g kg DM) and crude protein (CP) (± 40 g kg DM), as observed by Sá et al. (2020); Santos et al. (2020); Brito et al. (2020); Paulino et al. (2021); Pereira et al. (2021); Pastorelli et al. (2022); Sá et al. (2023); Soares et al. (2024), which requires care when formulating diets aimed at optimizing the use of this forage within the system.

Thus, the use of high proportions or as the only source of forage in the diet can cause nutritional disorders, causing liquefied feces, given that its fiber concentration is not sufficient to maintain adequate rumen functions (Rodrigues et al. 2016). Recent research has revealed that the lack of physically effective fiber in diets with high proportions of forage cactus alone has not been able to explain the occurrence of liquefied feces in ruminants (Cordova-Torres et al. 2017; de Barros et al. 2018).

According to Paulino et al. (2021), the manifestation of diarrhea in ruminants fed high proportions of cactus in the diet may be associated with poor hygiene during the feeding management of these animals, which increases contamination and the ingestion of groups of pathogenic bacteria via feed. Santos et al. (2022), when assessing the dynamics of the bacterial community in different mixtures of forage cactus and buffel grass hay (20, 60, 80, 90 and 100% forage cactus), as a function of exposure time to air (0, 3, 6, 12 and 24 h), observed an increase in the population of enterobacteria, especially *Escherichia coli*, after 6 h of exposure. This bacterium is mainly responsible for causing liquefied feces in animals, which is evidence of the reduction in the hygienic quality of the mixtures, probably due to the high levels of NFC present in forage cactus, aggravated by prolonged exposure to air.

In this context, the objective was to determine the optimum level of buffel grass hay as a source of physically effective fiber in diets with high proportions of forage cactus on the bioeconomic performance of sheep finished in a dryland feedlot.

## Materials and methods

This study was submitted to the Animal Research Ethics Committee of the Federal University of Paraíba (UFPB) and was approved under opinion n° 5391070619. All ethical principles and procedures relating to animal experimentation were strictly followed.

### Location, characterization, and duration of the experimental trials

The experimental trials with the animals were carried out on a property located in the municipality of São José dos Cordeiros, Paraíba, Brazil, during September and November 2020, totaling 51 days, the first 10 of which were the period of adaptation to the diets.

The municipality is in the Cariri Paraibano region, the Cariri Ocidental micro-region, characterized by a Bsh-type climate, with high temperatures (annual average of around 26 °C) and a low annual temperature range, rainfall with historical annual averages of less than 400 mm, whose rains are scarce, very concentrated over time, and irregular. Average sunshine is 2,800 h/year, relative humidity is around 50%, and average evaporation rates are around 2,000 mm/year (Alvares et al. 2013).

### Animals, experimental trials, and sampling

For the conduct of the experiment, 40 non-castrated male sheep, without a defined breed standard, aged between 6 and 7 months and with an initial body weight of 18.0 ± 2.6 kg, were selected. Before the beginning of the experimental period, the animals were weighed, vaccinated against clostridial diseases with Bovilis^®^ Poli-Star^®^ T (2 mL/animal, subcutaneously), and subjected to a health management protocol, which included treatment for endoparasites with albendazole (Valbazen^®^, 10 mg/kg body weight) and treatment for ectoparasites with 1% injectable ivermectin (Ivomec^®^ Plus, 0.2 mg/kg body weight). The sheep were housed in individual pens of approximately 3 m², equipped with a feed trough for providing the experimental diets and a water trough with unrestricted access to water.

There was a ten-day period of adaptation to the facilities and diets before the actual start of the experiment for data collection, which lasted 50 days, so the total duration was 60 days. The rations used consisted of Mexican elephant forage cactus (*Opuntia stricta Haw*) and buffel grass hay (*Cenchrus ciliaris* L.) harvested after one year of planting (Table 1). Both the buffel grass hay and the forage cactus were previously processed in a stationary forage machine (MC1001N Laboremus^®^), adjusted to achieve a particle size of approximately 1 cm for the hay and 2 cm for the cactus.

**Table 1:** Chemical composition of ingredients used in experimental diets based on dry matter

Items	Ingredients					
	Forage cactus	Buffel grass hay	Ground corn	Soybean meal	Cottonseed cake	Urea
Dry matter, g kg	123.0	913.5	892.4	906.0	927.0	975.3
Crude protein, g kg DM	44.0	38.3	94.7	400.0	202.7	281.0
Non-fibrous carbohydrates, g kg DM	572.9	139.3	710.0	300.0	175.0	0.0
Ether extract, g kg DM	19.3	10.8	66.0	17.1	94.3	0.0
Ash, g kg DM	118.0	79.2	16.4	64.8	46.8	2.1
Neutral detergent fiber, g kg DM	299.8	821.2	175.1	175.8	507.7	0.0
Acid detergent fiber, g kg DM	184.2	486.0	38.9	86.6	276.8	0.0
Cellulose, g kg DM	141.3	376.1	34.3	81.8	245.5	0.0
Hemicellulose, g kg DM	115.1	302.8	90.0	69.3	207.3	0.0
Lignin, g kg DM	45.4	79.7	12.1	15.6	41.8	0.0

The animals were allocated to a completely randomized design with four treatments and 10 repetitions. Four diets were examined, each composed of different proportions of buffel grass hay in the dry matter of the feed: 7.5% BF = 7.5% buffel grass hay; 15% BF = 15% buffel grass hay; 30% BF = 30% buffel grass hay, and 45% BF = 45% buffel grass hay on a dry matter (DM) basis. These diets were formulated to be isonitrogenous, meeting the requirements of the NRC guidelines (2007) for sheep weighing 18 kg and with a daily weight gain of 200 g (Table 2).

**Table 2:** Ingredient proportions and bromatological composition of experimental diets used to feed sheep different levels of Buffel grass hay on a dry matter basis

Items	^1^Buffel grass hay levels			
	45%	30%	15%	7.5%
Forage cactus, g kg	45.8	191.2	336.6	409.30
Buffel grass hay, g kg	436.2	290.8	145.4	72.70
Soybean meal, g kg	64.6	64.6	64.6	64.60
Ground corn, g kg	235.6	235.6	235.6	235.6
Cottonseed cake, g kg	178.0	178.1	178.3	178.4
Urea, g kg	9.2	9.00	8.9	8.8
Mineral mix, g kg	16.2	16.2	16.2	16.2
Ammonium chloride, g kg	13.5	13.5	13.5	13.5
Ammonium sulfate, g kg	1.0	1.0	1.0	1.0
Chemical composition				
Dry matter, g kg	703.0	406.2	285.6	248.7
Crude protein, g kg DM	142.3	141.7	142.0	141.3
Non-fibrous carbohydrates, g kg DM	273.7	336.7	399.8	431.3
Ether extract, g kg DM	39.0	40.3	41.5	42.2
Ash, g kg DM	20.4	31.8	43.2	48.9
Neutral detergent fiber, g kg DM	514.9	439.2	363.4	325.6
^2^Forage cactus: Buffel grass hay: Concentrate				
Forage cactus	35.2	72.9	85.3	88.8
Buffel grass hay	30.0	9.9	3.3	1.4
Concentrate	34.8	17.2	11.5	9.8
Ammonium chloride	0.06	0.03	0.02	0.01

^1^Levels in dry matter; ^2^Percentage ratio

The feed was offered *ad libitum* twice a day, in the same quantities at 8 am and 4 pm, weighed, and adjusted with a leftover margin of up to 10%. The leftovers were weighed daily to monitor the animals’ consumption of dry matter and other nutrients, and collected before a new feed was given, both in the morning and in the afternoon.

Dry matter (DM) consumption was monitored by weighing the amount of food offered and the leftovers daily during the 15th and 49th days of the experimental period. Dry matter intake was obtained by subtracting feed intake from leftovers, both based on DM. Samples of feed, diets, and leftovers were collected weekly to determine dry matter concentration.

The bromatological analyses of the ingredients and feed were carried out at the food analysis laboratory of the Instituto Nacional do Semiárido, Campina Grande-PB. Determinations included values for dry matter (DM; method 934.01), mineral matter (MM; method 942.05), crude protein (CP; method 954.01), ether extract (EE; method 920.39) and lignin (method 973.18), according to the Association of Official Analytical Chemists - AOAC (2000).

Neutral detergent fiber (NDF) and Acid detergent fiber (ADF) were determined according to the methodology proposed by Van Soest et al. (1991), using the ANKOM fiber analyzer (ANKOM200 Fibre Analyzer - ANKOM Technology Corporation, Fairport, NY, USA).

The concentration of non-fiber carbohydrates (NFC) was calculated based on the equations recommended by Sniffen et al. (1992) for food and by Hall (2000) for feed: NFC food = 100 - (%CP + %NDFap + %EE + %MM); and NFC fractions = 100 - [%NDFap + %EE + %MM + (%CP - %CPurea + %urea).

### Consumption and digestibility of dry matter and nutrients

The consumption of dry matter (DM) and other nutrients was monitored by weighing the feed and leftovers every day during both feeding shifts. In this way, the voluntary intake of dry matter and nutrients was determined by the difference between the amount of food offered and the leftovers. Nutrient intake was then converted from g kg to percentage.

Water intake (WI) was measured in the last 15 days of the experimental period, with water being weighed at the time of feeding and 24 h later. The evaporation rate was measured using four buckets positioned on the sides and one in the center of the shed. This made it possible to obtain the water intake via feed, drinker, and total.

After 19 days of the experimental period, the digestibility test was carried out, and samples of feed, leftovers, and feces were collected. Feces were collected from the end of the rectum on the 20th day (6 am and 2 pm), 21st day (8 am and 4 pm), and 22nd day (10 am and 6 pm).

The samples were weighed, identified and stored at -15 °C, then homogenized (making up one composite sample per animal) and pre-dried in an oven with forced ventilation at 65 °C for 72 h, after which the samples were quantified in terms of DM, CP, ADF and lignin according to methods also described by National Semiarid Institute. The NDF evaluations followed protocols suggested by Van Soest et al. (1991).

To assess the indigestible components, the methodology proposed by Casali et al. (2008) was used, where the samples were packed in non-woven fabric bags (4 × 5 cm) in triplicate and incubated for 288 h in the rumen of a Girolando male bovine with an average live weight of 500 kg.

### Productive performance

On the first day of the experimental period, a 16-hour solid fast was carried out to quantify the initial weight (IW). Weigh-ins without fasting were carried out every two weeks (2 weigh-ins) to monitor the animals’ development. On the 44th day of the experimental period, another weighing was carried out with a previous solid fast of 16 h, resulting in the final weight (FW).

The total gain (TG) was determined by subtracting the FW from the IW. The average daily gain (ADG) was calculated by dividing the TG by the total duration of the experimental period, which was 54 days. feed conversion (FC) was obtained by dividing average dry matter intake (DMI) by ADG. Finally, feed efficiency (FE) was calculated by dividing ADG by DMI.

### Economic evaluation

To carry out the economic analysis of the feed provided, the market prices of the feed ingredients and the live weight of the animals sold at the end of the experimental period were taken into account. Based on the cost of each feed and its dry matter consumption, the economic result generated by each experimental diet was calculated.

Considering the prices practiced during the experiment in October/November 2020, the following values were adopted: US$ 3.36 per kg^− 1^ of live weight of the rams at the end for slaughter; US$ 36.36 per animal for acquisition at the beginning of the experiment; US$ 0.42 per kg^-1^ of soybean meal; US$ 0.15 per kg^− 1^ of corn meal; US$ 0.17 per kg^− 1^ of cottonseed cake; US$ 0.36 per kg^− 1^ of urea; US$ 0.07 per kg^− 1^ of mineral nucleus; US$ 0.22 per kg^− 1^ of buffel grass hay; and US$ 0.02 per kg^− 1^ of forage cactus.

In addition to the experiment, extra expenses were incurred, including US$ 45.45 in veterinary products, US$ 27.27 in machine maintenance and general tools, as well as a labor cost of US$ 263.55.

### Statistical analysis

The effect of the levels of buffel grass on the diet on consumption, digestibility, and animal performance was verified by regression analysis, using the standard error of the mean (SEM) and the significance of the regression parameters as criteria for choosing the models, tested by the t-test at a 5% significance level.

The following mathematical model was used: $\mathrm{Yi}=\beta 0+\beta1\mathrm{Xi}+\mathrm{ei}$

Where:

Υi = observed value for the dependent variable Y at the i-th level of the independent variable X;

β0 = regression constant. Represents the intercept of the Y axis;

β1 = regression coefficient. Represents the variation of Y as a function of the variation of one unit of variable X;

Xi = i-th level of the independent variable X (i = 1,2, …, n);

ei = the error associated with the distance between the observed value Υi and the corresponding point on the curve of the proposed model for the same level i of X.

## Results

### Animal water intake

There was a quadratic effect (*Q* < 0.001) for water consumption via feed, as a function of the levels of buffel grass hay, with maximum consumption estimated by the regression model at a level of 25% buffel grass hay. A linear effect *(L* < 0.001) . However, a quadratic effect (Q < 0.001) was observed for total water consumption, with maximum consumption estimated by the regression model at the 35% level of buffel grass hay (Table 3).

**Table 3:** Water intake in feedlot sheep fed diets based on forage cactus with varying levels of Buffel grass hay

Water intake (L/day)	^1^Buffel grass hay levels				^2^SEM	^3^Effects	
	45%	30%	15%	7.50%		L	Q
Via feed	1.289	2.320	2.398	2.076	0.610	< 0.001	< 0.001
Via drinking trough	2.745	1.979	1.358	0.881	0.860	< 0.001	0.857
Total	4.034	4.299	3.756	2.954	1.048	< 0.001	< 0.001

^1^Levels based on dry matter; ^2^SEM: Standard error of the mean; ^3^L: linear effect; Q: quadratic effect

### Productive performance

A quadratic effect (*p* < 0,05) was observed for OMI (*Q* < 0.001), DMI %BW^0.75^ (*Q* < 0.001), DMI (*Q* < 0.001), CPI (*Q* < 0.001), EEI (*Q* < 0.001), NDFI (*Q* < 0.001), NFCI (*Q* < 0.001), TCHI (*Q* < 0. 001), TDNI (*Q* < 0.001) and MEI (*Q* < 0.001), with maximum intakes estimated at 35, 23, 42, 23, 20, 25, 10, 25, 25 and 24% of buffel grass hay, respectively (Table 4).

**Table 4:** Nutrient intake in feedlot sheep fed with levels of Buffel grass hay in diets based on forage cactus

Variables	^1^Buffel grass hay levels				^2^SEM	^3^Effects	
	45%	30%	15%	7.50%		L	Q
^4^OMI, g kg DM	0.944	1.414	1.259	0.993	0.323	0.220	< 0.001
^5^DMI, %BW^0,75^	3.48	4.85	4.67	3.89	0.137	0.005	< 0.001
^6^DMI, g kg	1.083	1.678	1.528	1.183	0.391	0.027	< 0.001
^7^CPI, g kg DM	0.206	0.362	0.395	0.341	0.012	< 0.001	< 0.001
^8^EEI, g kg DM	0.061	0.110	0.116	0.110	0.030	< 0.001	< 0.001
^9^NDFI, g kg DM	0.677	1.044	0.936	0.730	0.244	0.070	< 0.001
^10^NFCI, g kg DM	0.281	0.674	0.899	0.873	0.219	< 0.001	< 0.001
^11^TCHI, g kg DM	0.863	1.551	1.736	1.505	0.447	< 0.001	< 0.001
^12^TDNI, g kg DM	0.882	1.627	1.868	1.657	0.411	< 0.001	< 0.001
^13^MEI, Mcal kg	3.891	6.201	6.070	4.777	1.453	< 0.001	< 0.001

^1^Dry matter levels; ^2^SEM: Standard error of the mean; ^3^L: linear effect; Q: quadratic effect. OMI: Organic matter intake; DMI (% BW^0.75^): Dry matter intake based on body weight; DMI: Dry matter intake; CPI: Crude protein intake; EEI: Ether extract intake; NDFI: Neutral detergent fiber intake; NFCI: Non-fibrous carbohydrate intake; TCHI: Total carbohydrate intake; TDNI: Total digestible nutrient intake; MEI: Metabolizable energy intake

For the apparent digestibility variables, there was a linear effect for OMD (*L* < 0.001), DMD (*L* < 0.001), CPD *(L* < 0.001), EED (*L* < 0.001), NDFD (*L* < 0.001), NFCD (*L* < 0.001) and CHID (*L* < 0.001), as a function of the levels of buffel grass hay in the diet, ranging from 741.34 to 868.87 g kg, 654.04 to 725.58 g kg, 697.22 to 756.02 g kg, 827.87 to 784.73 g kg, 560.90 to 571.74 g kg, 872.90 to 930.38 g kg and 602.07 to 751.12 g kg, respectively (Table 5).

**Table 5:** Apparent digestibility of nutrients in feedlot sheep fed with different levels of Buffel grass hay in diets based on forage cactus

Variables	^1^Buffel grass hay levels				^2^SEM	^3^Effects	
	45%	30%	15%	7.5%		L	Q
^4^OMD, g kg DM	741.34	790.26	848.39	868.87	30.78	< 0.001	0.997
^5^DMD, g kg	654.04	668.36	712.59	725.58	41.09	< 0.001	0.024
^6^CPD, g kg DM	697.22	702.70	741.08	756.02	52.52	< 0.001	0.013
^7^EED, g kg DM	827.87	813.49	759.43	784.73	50.79	< 0.001	0.138
^8^NDFD, g kg DM	560.90	552.69	595.33	571.74	65.30	< 0.004	0.844
^9^NFCD, g kg DM	872.90	886.30	913.86	930.38	43.87	< 0.001	0.073
^10^CHID, g kg DM	602.07	651.50	736.11	751.12	63.03	< 0.001	0.662

^1^Dry matter levels; ^2^SEM: Standard error of the mean; ^3^L: linear effect; Q: quadratic effect

OMD: Organic matter digestibility; DMD: Dry matter digestibility; CPD: Crude protein digestibility; EED: Ether extract digestibility; NDFD: Neutral detergent fiber digestibility; NFCD: Non-fibrous carbohydrate digestibility; CHID: Total carbohydrate digestibility

There was a quadratic effect for FW (*Q* = 0.041), TG (*Q* < 0.001) and ADG (*Q* < 0.001), as a function of the levels of buffel grass hay, with the maximum gains estimated by the regression model at levels of 27, 27 and 28% buffel grass hay, respectively. However, there was no effect (*p* > 0.05) for AC and AS, with overall averages of 7.57 kg DM and 138.5 g DM, respectively (Table 6).

**Table 6:** Performance of feedlot sheep fed with levels of Buffel grass hay in diets based on forage cactus

Variables	^1^Buffel grass hay levels				^2^SEM	^3^Effects	
	45%	30%	15%	7.5%		L	Q
Final weight, kg	31.2	34.82	32.8	30.62	1.489	0.728	0.041
Total gain, kg	7.37	10.05	9.34	6.89	0.546	0.762	< 0.001
^4^ADG, g dia	168.0	229.0	212.0	156.0	0.012	0.754	< 0.001
^5^FCR, kg DM	6.84	7.49	7.56	8.38	0.650	0.132	0.859
^6^FE, g DM	153.0	135.0	137.0	129.0	0.006	0.106	0.639

^1^Dry matter levels; ^2^SEM: Standard error of the mean; ^3^L: linear effect; Q: quadratic effect

^4^ADG: average daily gain; ^5^FCR: feed conversion ratio; ^6^FE: feed efficiency

### Economic indices of the feedlot

The average cost per kg of concentrate was US$ 0.17 for all the levels evaluated. The total cost of forage cactus increased as the levels of buffel grass hay in the diet decreased (15 and 7.5%), while the opposite result was observed for the total costs of buffel grass hay (45 and 30%). Concentrate and feed costs were higher at the 45 and 30% levels of buffel grass hay (Table 7).

**Table 7:** Economic evaluation of confined sheep fed diets based on forage cactus

Variables	^1^Buffel grass hay levels			
	45%	30%	15%	7.5%
Cost/kg of concentrate (US$)	0.17	0.17	0.17	0.17
Total cost with forage cactus (US$)	5.20	29.27	27.24	27.74
Total cost with buffel grass hay (US$)	53.22	31.40	12.65	5.25
Total cost with concentrate (US$)	48.74	43.28	36.73	29.10
Total cost with feeding (US$)	107.17	93.95	74.81	62.09
Animal acquisition cost (US$)	363.64	363.64	363.64	363.64
Total operational cost (US$)	84.07	84.07	84.07	84.07
Gross revenue (US$)	1024.83	1096.31	1065.47	974.45
Net revenue (US$)	469.96	554.67	541.15	464.65

Animal acquisition and total operating costs did not differ between the levels of buffel grass hay in the diets, with average costs of US$ 363.64 and US$ 84.07, respectively. However, gross and net revenues were higher at the 30 and 15% levels of buffel grass hay in the diet (Table 7).

The regression equations for all variables measured in confined sheep fed diets with high proportions of forage cactus containing levels of buffel grass hay are presented in Table 8.

**Table 8:** Regression equations for variables measured in feedlot sheep fed diets with different levels of Buffel grass hay

Variables	Equation	*R*²
Water intake via feed	Ŷ = 1.52 + 0.10x – 0.002x²	0.9999
Water intake via drinking trough	Ŷ = 0.56 + 0.05x	0.9959
Total water intake	Ŷ = 2.05 + 0.14x – 0.002x²	0.9919
Organic matter intake	Ŷ = 0.55 + 0.07x – 0.001x²	0.9977
Dry matter intake relative to metabolic weight (DMI %BW⁰·⁷⁵)	Ŷ = 2.77 + 0.18x – 0.004x²	0.9973
Dry matter intake	Ŷ = 0.65 + 0.083x – 0.001x²	0.9994
Crude protein intake	Ŷ = 0.26 + 0.014x – 0.0003x²	0.9968
Ether extract intake	Ŷ = 0.08 + 0.004x – 0.0001x²	0.9994
Neutral detergent fiber intake	Ŷ = 0.39 + 0.05x – 0.001x²	0.9988
Non-fibrous carbohydrate intake	Ŷ = 0.84 + 0.01x – 0.0005x²	0.9963
Total carbohydrate intake	Ŷ = 1.20 + 0.05x – 0.001x²	0.9934
Total digestible nutrients intake	Ŷ = 1.38 + 0.05x – 0.001x²	0.9941
Metabolizable energy intake	Ŷ = 3.01 + 0.29x – 0.006x²	0.9957
Organic matter digestibility	Ŷ = 896.84–3.47x	0.9977
Dry matter digestibility	Ŷ = 739.37–2.02x	0.9566
Crude protein digestibility	Ŷ = 764.78–1.64x	0.9129
Ether extract digestibility	Ŷ = 758.51 + 1.55x	0.7187
Neutral detergent fiber digestibility	Ŷ = 586.38–0.67x	0.3575
Non-fibrous carbohydrate digestibility	Ŷ = 938.43–1.54x	0.9686
Total carbohydrate digestibility	Ŷ = 787.85–4.21x	0.9819
Final body weight	Ŷ = 26.63 + 0.59x – 0.011x²	0.9862
Total weight gain	Ŷ = 3.95 + 0.48x – 0.009x²	0.9843
Average daily gain	Ŷ = 0.07 + 0.011x – 0.0002x²	0.9839

## Discussion

The water intake via feed (WIF) of 2.32 L/day recorded in the diet with 30% buffel grass hay inclusion (Table 3) was directly influenced by the DMI of 1.678 g/kg observed in animals consuming this diet (Table 4).

There is a positive relationship between DMI and WI, altered only when factors such as increased temperature, CP content, and dietary salt are elevated (Teixeira et al. 2006). In this context, at higher temperatures, as observed in semi-arid regions, it is normal for animals to increase their water intake.

Due to the high water retention capacity in semi-arid regions (Consoli et al. 2013; Fonseca et al. 2019), forage cactus is traditionally used in diets to meet the animals’ water requirements, and its ability to increase water intake via feed is well documented (Mayer and Cushman 2019; Nipane et al. 2021; Pastorelli et al. 2022).

The results of this experiment, showing a reduction in water intake from the drinking trough and an increase in water intake via feed in animals fed diets with higher proportions of forage cactus, corroborate findings from other studies that observed similar patterns (Andrade-Montemayor et al. 2011; Cordova-Torres et al. 2022; da Silva et al. 2021, 2024; Rezende et al. 2020; Tegegne et al. 2007).

In the diets with 15% and 30% buffel grass hay inclusion, the higher DMI recorded directly influenced the greater intake of other nutrients. Furthermore, the intake values for all variables in these diets were similar (Table 4).

All intake variables exhibited a positive quadratic effect. Determining the maximum point revealed that a 25% buffel grass hay inclusion level would maximize nutrient intake, including DMI (%BW^0.75^), CPI, NDFI, TCHI, TDNI g/kg, and MEI Mcal kg.

On the other hand, CPI, EEI, NFCI, TCHI, TDNI g kg, and MEI Mcal kg also showed a linear effect along with the quadratic effect, where EEI g/kg, with a maximum point at 20% buffel grass hay inclusion, showed oscillations between 7.5% and 15% inclusion, and a reduction in EEI values when the inclusion reached 45% (Table 4).

The bromatological analysis of ingredients used in the diets revealed that forage cactus contains almost twice the EE compared to buffel grass hay (Table 1), which likely explains the lower EEI observed when buffel grass hay inclusion increased.

The lowest DMIs (1.183 g kg and 1.083 g kg) were recorded (Table 4) when the feed contained 7.5% and 45% buffel grass hay inclusion, respectively, representing the highest and lowest forage cactus proportions (Table 2). The average dry matter content of forage cactus used in this experiment was 123 g kg DM (Table 1). Therefore, the high moisture content explains why animals fed diets with lower buffel grass hay inclusion had lower DMI (Table 4).

Conversely, the bromatological evaluation found NDF contents of 821.2 g kg DM for buffel grass hay, and EE contents of 66 g kg, 17.1 g kg, and 94.3 g kg DM for corn meal, soybean meal, and cottonseed meal, respectively (Table 1), along with a higher concentrate proportion in this diet (Table 2).

Thus, the higher NDF and ether extract contents in the diet with 45% buffel grass hay inclusion led to faster satiety in animals and limited DMI. According to Inácio et al. (2019), DMI reduction can result from increased dietary fat inclusion, as well as elevated NDF, which prolongs rumination time and slows gastrointestinal transit.

Although EEI and NDFI were lower for animals fed the diet with 45% buffel grass hay inclusion compared to those fed diets with 15% and 30% buffel grass hay inclusion, this condition could be attributed to the high acceptance of forage cactus by sheep (Fotius et al. 2014; Magalhães et al. 2021), as the cactus proportions were higher in these diets compared to the 45% buffel grass hay inclusion diet (Table 2).

The DMI results are similar to those found in other studies where DMI increased when animals consumed diets with lower proportions of forage cactus (Tegegne et al. 2007; Vieira et al. 2008).

However, they differ from other results where animals fed diets with higher proportions of forage cactus showed increased DMI (Gama et al. 2021; Rezende et al. 2020; Siqueira et al. 2017), a factor attributed to the presence of NFC, which are rapidly degradable and stimulate passage rate through the gastrointestinal tract, enhancing intake.

The apparent digestibility showed a decreasing linear effect for OM, DM, CP, EE, NDF, NFC, and TCHI, decreasing as the inclusion of buffel grass hay increased (Table 5). Results similar to those of this experiment, where there was a reduction in the digestibility of DM and CP in animals fed diets with higher amounts of forage cactus, were observed by Tegegne et al. (2007), as well as the reduction of NDF digestibility reported by Lopes et al. (2020).

However, unlike what was verified in this experimental trial, the digestibility of DM, OM, NFC, and TCHI in animals fed higher quantities of forage cactus increased in other studies (Lopes et al. 2020; Rezende et al. 2020; Shruthilaya et al. 2022).

According to Lopes et al. (2020), different forage cactus genotypes promote different fermentation patterns. In this context, this could explain the differences in digestibility observed between those studies and the present one.

The diet with the highest proportion of forage cactus resulted in lower final weight (30.62 kg), total weight gain (6.89 kg), average daily gain (0.156 kg), feed efficiency (0.129 kg), and higher feed conversion ratio (8.38 kg), compared to the other diets, indicating inferior performance. Even so, the values obtained suggest the feasibility of using such a diet for sheep feeding, considering the cost of the diet with 7.5% buffel grass hay inclusion (Table 7).

The performance results are supported by the values found for DMI and OMI (Table 4), where the diets with 15% and 30% buffel grass hay inclusion had the highest values.

Other studies found better performance data as the amount of forage cactus in the diet increased, differing from the results found in this trial (Cordova-Torres et al. 2022; da Silva et al. 2021; Gebremariam et al. 2006).

On the other hand, the results from the study by Tosto et al. (2021) were similar, showing better performance with a diet containing a lower proportion of forage cactus, although nutrient digestibility and intake were lower in that diet.

Filho et al. (2022) evaluated the performance of feedlot-finished sheep fed with different levels of forage cactus cladodes and observed that lower proportions of forage cactus resulted in higher final, daily, and feed efficiency gains, findings like those observed in this study. However, unlike what was seen in the present trial, feed conversion ratio was improved as the proportion of forage cactus cladodes increased in Filho et al. (2022).

Despite the proportional cost increase with higher levels of buffel grass hay inclusion in the experimental diets, the performance data (Table 6) showed that feed efficiency and feed conversion ratio were impaired when the diets contained a higher proportion of forage cactus and a lower proportion of hay, directly affecting the final net revenues generated by these treatments. Thus, the treatment with 30% buffel grass hay inclusion showed the best balance between feed efficiency, feed conversion, diet cost, and net revenue compared to the other treatments.

All experimental diets generated positive net revenues. It was possible to observe the formation of two close groups: one including treatments with 88.78% and 35.22% forage cactus proportion, with net revenues of US$ 464.65 and US$ 469.96, respectively, and another group with treatments with 85.25% and 72.86% forage cactus proportion, with net revenues of US$ 541.15 and US$ 554.67, respectively.

These data show that all treatments can be adopted depending on the producer’s situation and the resources available, once again emphasizing the need for careful feeding management when adopting diets with a high proportion of forage cactus.

## Conclusion

Based on productive performance and the net revenue obtained in the feedlot, the inclusion of 30% buffel grass hay in diets with high proportions of forage cactus is recommended for feedlot sheep.

## Data Availability

The data supporting the conclusions of this study are available upon reasonable request to the corresponding author.
